# Telemedicine’s Potential to Support Good Dying in Nigeria: A Qualitative Study

**DOI:** 10.1371/journal.pone.0126820

**Published:** 2015-06-01

**Authors:** Jelle van Gurp, Olaitan Soyannwo, Kehinde Odebunmi, Simpa Dania, Martine van Selm, Evert van Leeuwen, Kris Vissers, Jeroen Hasselaar

**Affiliations:** 1 Department of Anesthesiology, Pain, and Palliative Medicine, Radboud University Medical Center, Nijmegen, Netherlands; 2 Centre for Palliative Care Nigeria and Hospice and Palliative Care Unit, University College Hospital, Ibadan, Nigeria; 3 Hospice and Palliative Care Unit, University College Hospital, Ibadan, Nigeria; 4 Department of Telemedicine, University College Hospital, Ibadan, Nigeria; 5 Amsterdam School of Communication Research, University of Amsterdam, Amsterdam, Netherlands; 6 Department of IQ Healthcare, Ethics Section, Radboud University Medical Center, Nijmegen, Netherlands; University of Stirling, UNITED KINGDOM

## Abstract

**Objectives:**

This qualitative study explores Nigerian health care professionals’ concepts of good dying/a good death and how telemedicine technologies and services would fit the current Nigerian palliative care practice.

**Materials and Methods:**

Supported by the Centre for Palliative Care Nigeria (CPCN) and the University College Hospital (UCH) in Ibadan, Nigeria, the authors organized three focus groups with Nigerian health care professionals interested in palliative care, unstructured interviews with key role players for palliative care and representatives of telecom companies, and field visits to primary, secondary and tertiary healthcare clinics that provided palliative care. Data analysis consisted of open coding, constant comparison, diagramming of categorizations and relations, and extensive member checks.

**Results:**

The focus group participants classified good dying into 2 domains: a feeling of *completion of the individual life* and *dying within the community*. Reported barriers to palliative care provision were socio-economic consequences of being seriously ill, taboos on dying and being ill, restricted access to adequate medical–technical care, equation of religion with medicine, and the faulty implementation of palliative care policy by government. The addition of telemedicine to Nigeria’s palliative care practice appears problematic, due to irregular bandwidth, poor network coverage, and unstable power supply obstructing interactivity and access to information. However, a tele-education ‘lite’ scenario seemed viable in Nigeria, wherein low-tech educational networks are central that build on non-synchronous online communication.

**Discussion:**

Nigerian health care professionals’ concepts on good dying/a good death and barriers and opportunities for palliative care provision were, for the greater part, similar to prior findings from other studies in Africa. Information for and education of patient, family, and community are essential to further improve palliative care in Africa. Telemedicine can only help if low-tech solutions are applied that work around network coverage problems by focusing on non-synchronous online communication.

## Introduction

The WHO estimates that in Africa each year approximately 961.000 adults and 537.000 children are in need of palliative care at the end of life [1]. Realization of palliative care’s internationally recognized core values—to improve the quality of life of patients and their relatives facing a life threatening illness by early identification and impeccable assessment of pain and other problems—would strongly benefit these patients [2–6]. In general, African patients who require palliative care suffer from treatable but persistent infectious diseases (e.g., HIV/AIDS, severe tuberculosis) and/or non-communicable diseases (e.g., cancer). A substantial part of these patients are young children [7]. Although palliative care’s values are internationally recognized, a socio-cultural context defines the final interpretation and realization of these values. The WHO, anticipating on this ‘couleur locale’, focuses on a ‘community health approach’ to improve palliative care in Africa [8]. Different projects stimulate ‘team development and networking with community-focused action’, training and education, and integration of care with a special emphasis on home-based care.

Although palliative care in sub-Saharan Africa is gaining quality thanks to increasing professional attention [9], it mainly remains a family affair [10]. Currently, the economic burden for most families is substantial because providing care limits labor activities. Moreover, both a patient’s suffering and the burden of care increase when social exclusion inspired by fears of a patient’s disease occurs [10,11]. As the potential social isolation, the costs of fees for treatment, and transportation time limit patients’ follow up, patients often become underserved [7,12].

Faulty community knowledge about serious illnesses obstructs early self or peer diagnosis and timely referral to professional care. As a consequence, patients often arrive at the hospital with seriously advanced diseases, leaving medical specialists the sole option of palliation [6,13,14]. This perceived inability to cure harms the community’s trust in ‘Western medicine’ and paves the way for alternative, traditional treatments [13].

Notwithstanding poverty, an increasing number of Africans use mobile phones [15]. Experiments with mobile phones, texting and help lines for cancer and HIV care in Nigeria and Kenya have shown positive outcomes [12,16]. Mobile health (mHealth; e.g., text messaging, mobile phone reminders; see [17]) has been used in low income countries to assist patients and families with keeping follow-up appointments, adhering to treatments, experiencing support to initiate consultation themselves, and to promote health in the community [12,16–19]. In general, information and communication technologies seem supportive for health care professionals in developing countries. They can function as decision support systems or hubs to web-based information [17,18,20], as asynchronous or real-time web-based learning media for continuous training [17,21], or as instruments (e.g., e-mail and videoconferencing) to build national and international consultation networks [17,21–23].

In Western countries, mobile communication technologies are increasingly applied in palliative care [24,25]. The Radboud University Medical Centre, Netherlands, currently implements and investigates an interactive teleconsultation service in palliative homecare [24]. In addition to this research in an European context, the Expertise Centre for Palliative Care (Radboudumc, Nijmegen, Netherlands) and the Centre for Palliative Care Nigeria (CPCN, Ibadan) intended to explore the potential fit of telemedicine technologies and services (e.g., monitoring, education, consultation) for palliative care in African countries where poverty, limited access to health care, long distance travelling, and different perspectives on good dying and death make up for a different care context. To be sustainable in Africa, the ICTs and the offered services have to make sense to African societies in general and local communities in particular [21,26,27]. Ideally, telemedicine is developed bottom up, contains locally relevant information, and enables locally meaningful interactions [19,22,28–30]. This study’s objectives were twofold: first, we aimed to conceptualize good dying and/or a good death as defined by Nigerian health care professionals working in the field of palliative care. Second, we set out to explore how telemedicine technologies and services would fit the palliative care practice as described by the Nigerian health care professionals, aiming for recommendations for the implementation of telemedicine in Nigerian palliative care.

## Study Design

We performed a qualitative study of a) perceptions on good dying/a good death of Nigerian professionals working in palliative care and b) the opportunities and barriers for including telemedicine into Nigerian palliative care practices in order to theorize about and model a potential telemedicine intervention in such a complex palliative care practice [31].

### Ethics Statement

The University of Ibadan/University College Hospital Health Research Ethics Committee, Nigeria, approved the research proposal (file number: NHREC/05/01/2008a).

Focus group participants all provided written informed consent. Field visits took place under the auspices of Nigerian palliative care physicians who gathered verbal informed consent before introducing the foreign researchers. As the researchers did not engage in any medical treatment or direct patient-researcher interaction, verbal informed consent was considered sufficient. Health care experts’ and telecommunication experts’ verbal agreement to participation in interviews was equally considered sufficient. Representatives of CPCN/UCH always accompanied the researchers during the interviews and can confirm the verbal consent.

The Health Research Ethics Committee approved this consent procedure.

### Location of Study

Nigeria is the most populous nation in Africa, with a positive GDP growth mainly driven by its large oil industry. However, large health disparities exist as many citizens live in poverty and lack access to health care [32]. In Nigeria, a considerable number of the estimated total population of 167 million suffers from life-threatening chronic illnesses (e.g., 377 per 100.000—age 30–70—die of cardiovascular disease and diabetes [33]; approximately 210.000 people die annually of HIV/Aids (statistics of 2011) [34] and at least 71.600 of cancer (statistics of 2012) [13,14,35]). The Nigerian government funds approximately 31% of the total health expenditure [33]. With health care insurances largely absent for the majority of the Nigerian population, the remaining costs for health care have to be paid out-of-pocket by its users [36].

Ibadan is the third largest metropolitan area in Nigeria, after Lagos and Kano, with a population of 1,338,659 according to a 2006 census. UCH is one of the few hospitals in Nigeria that can provide tertiary care such as chemotherapy and radiation. As a consequence, this hospital attracts people suffering from cancer living within a radius up to 500 km around Ibadan. The Centre for Palliative Care Nigeria (CPCN), a non-profit organization stimulating and supporting palliative care initiatives in Nigeria, established a palliative care team for palliative patients in the University College Hospital in Ibadan as well as for palliative outpatients who reside within a 30 km-radius around Ibadan. However, according to CPCN about half of the palliative patients leaving UCH live outside this 30 km-radius and are therefore deprived of continuous, adequate palliative outpatient care. UCH has a telemedicine unit that used to be the operating base for a medically equipped bus with satellite technologies for real time teleconsultations and currently functions as the base for a Pan African-India telemedicine project for educational purposes and international teleconsultations. The experiment with the bus had to stop due to failing satellite connections and lack of funding.

Two Dutch researchers (JG; JH) made a 10 days site visit to the CPCN in January 2013. During the time of the visit, CPCN hosted a national five weeks palliative care initiators course for Nigerian health care professionals.

### Participant Selection

Prior to the study, initial relationships were established between the research team of RadboudUMC Palliative Care Centre [JG, JH] and representatives of CPCN [OS, KO, SD], resulting in a co-authored research proposal. This joint writing process already brought about a dialogue about different worldviews, socio-cultural structures, and allowed for distinguishing key role players in Nigerian palliative care.

In January 2013, CPCN’s representatives [OS, KO, SD] arranged three focus groups by using purposeful sampling: one focus group consisted of 5 members of CPCN’s palliative care team, two other focus groups each contained 8 Nigerian health care professionals attending a national palliative care initiators course in Ibadan, Nigeria. The study participants were drawn from multidisciplinary health professionals from nine different hospitals in Nigeria. Palliative care is a new area of health care being promoted in Nigeria by mostly anesthesiologists and oncologists (surgical, gynecological, medical) due to the pain and suffering they witness in their patients, most of whom present with late stages of cancer. However, pharmacists, nurses, and medical social workers also participated. Moreover, CPCN arranged interviews with key role players in palliative care within (the service area of) UCH and with representatives of Nigeria’s telecommunication structures (Table 1).

**Table 1:** Overview of study participants.

***Focus groups***	
Focus group 1—Palliative care team CPCN	
Nurse	2
Doctor (Medical officer)	3
Focus group 2—Attendees palliative care course Ibadan	
Nurse	4
Pharmacists	3
Doctor (Pediatrician)	1
Focus group 3—Attendees palliative care course Ibadan	
Nurse	1
Pharmacist	2
Doctor (Obstetrician gynecologist)	2
Doctor (Anesthesiologist)	1
Doctor (Surgeon)	1
Medical social worker	1
***Unstructured interviews***	
Key role players for palliative care within UCH	
Chief Medical Director	
Head of Department Family Medicine/PEPFAR	
Head of Department Radiotherapy	
Providers of the socio-technical context	
Representatives of UCH Telemedicine Unit	
Representative of zonal office Nigerian Communications Commission	
Representatives of telecom provider Glo Mobile	
***Field visits***	
Ward rounds UCH (tertiary)	
Visit to a palliative outpatient service in the Ibadan-area	
Visit to a private secondary care facility (urban)	
Visit to a Catholic, private secondary facility (sub-urban)	
Visit to a private primary/secondary facility (rural)	
Visit to a primary care clinic (urban)	

### Data collection

Three data collection methods were used throughout this research project. Focus groups with different Nigerian health care professionals were held to discover their perspectives on good dying and reaching a good death. In addition, unstructured interviews were conducted with the director of UCH and two physician directors to better understand the Nigerian health care system on a macro level. Finally, direct observations were applied to study the palliative care practices in various Nigerian health care contexts. In addition, unstructured interviews with representatives of the socio-technical context (UCH’s telemedicine unit; telecom providers; government) were conducted.

### Focus groups

Researchers JG (MSc; social sciences/ethicist; experienced qualitative researcher; male) and JH (PhD; health care policy/ethicist; assistant professor in palliative care; male), assisted by CPCN representative KO (MD; palliative care physician; female), moderated the 3 focus groups (FG 1–3). The first focus group took place at the CPCN headquarters, the other two at the location of the palliative care course in the city centre. The focus groups took, on average, 60 minutes.

A small literature review was performed by JG and JH to prepare for the focus groups. Central to the focus groups was an interview guide with four open-ended questions: (a) What do Nigerians generally consider to be a good death?, (b) What role/importance is ascribed to palliative care in Nigeria’s current health care structure?, (c) What role/importance do you ascribe to palliative care and why?, (d) What is the potential role of ICTs, and especially your mobile phone, in your daily work?

The focus groups started with participants writing down on separate cards all imaginable aspects of dying well/a good death in Nigeria and categorizing these cards. The investigators followed and stimulated the internal discussions concerning this categorization. The investigators used clarifying and probing questions where responses were not clearly understood. Using this first categorization, the moderators asked the participants to zoom in on those aspects that explained their professional contributions to the good dying of their patients and on the aspects of good dying/good death that were directly affected by Nigeria’s health care structures. Finally, the potential role of ICTs was discussed in the light of elaborated categorization.

Participants’ categorizations and explanations were recorded (photographs; audio recordings—essential fragments transcribed verbatim; field notes) and, afterwards, discussed with CPCN’s representative, physician KO.

#### Unstructured interviews with experts

The objectives of the unstructured interviews with experts were to explore the organization of health care, including palliative care, in the Ibadan-region as well as to explore the (potential) role of telecommunication in the Nigerian palliative care context. Extensive field notes were made during and after these interviews. These interviews were not audio-recorded except for one interview with members of the telemedicine unit of the University College Hospital. The duration of interviews felt within the range 30–50 minutes. Due to cultural differences, audio recording did not seem appropriate in these initial interviews. More established researcher-respondent relationships would likely provide room for audio recording.

#### Direct observations

Six field visits were conducted. During these visits, the focus was on observing and recording the various forms of palliative care provision in different health care facilities: a) tertiary, secondary, and primary care facilities, b) public and private health care facilities, and c) urban (Ibadan) and rural facilities (Table 1). Observations included conversations and discussions with staff members, accompanying physicians during ward rounds, and occasional, brief encounters with patients (who seldom spoke English). The direct observations were captured in extensive field notes to complement the findings from the focus groups and interviews.

### Data Analysis

CAQDAS ATLAS.ti software, version 6, was used for the data analysis.

#### Step 1. Data analysis during the site visit

As part of the focus groups, participants collected and categorized aspects concerning good dying and/or good death. To further establish respondent validation [37], this categorization and preliminary findings from the open interviews and observations were extensively discussed during “member checks” [38] with CPCN representatives to discover their meaning and significance.

#### Step 2. Data analysis after the site visit

After the site visit, researchers JG and JH performed a qualitative analysis on the available research material. The transcripts of the focus groups and the field notes of the open interviews and direct observations were pooled and submitted to open coding by JG. A ‘constant comparative analysis’ of codes and accompanying text segments from the transcripts was then used to build a tentative categorization of good dying/a good death in Nigeria, as well as a categorization of barriers and opportunities for telemedicine in a Nigerian palliative care context [38,39]. Conceptual mapping (called ‘network views’ in ATLAS.ti) was used to clarify relationships between (sub)codes [39]. The results of this qualitative analysis were compared to the results of the analysis during the site visit, and again further discussed by all authors during the co-writing process of this article.

## Results

**Fig 1:**
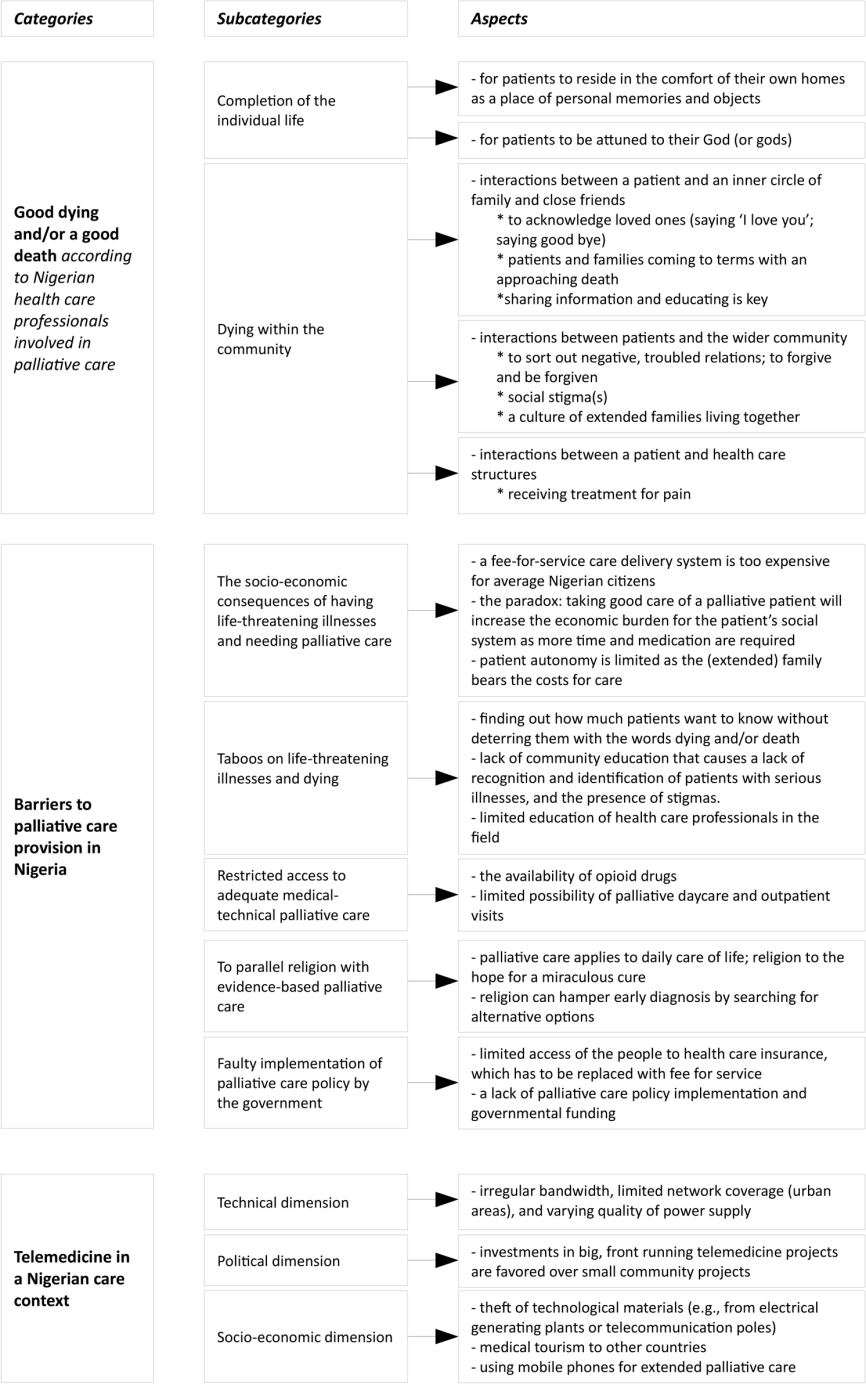
Schematic overview of categories and subcategories defining how telemedicine fits into Nigerian palliative care practice(s).

Analysis of the data from the focus groups, open interviews, and direct observations resulted in the following description of good dying/a good death in Nigeria according to Nigerian health care professionals involved with palliative care. In addition, study participants reported on barriers for palliative care in Nigeria. Finally, the data showed barriers and opportunities for telemedicine in this Nigerian palliative care context that guided an advice for any possible development and implementation of telecommunication in palliative care in Nigeria (Fig 1).

### Nigerian Conceptions of Good Dying and/or a Good Death

With regard to the participants’ conceptualization of good dying and/or a good death in Nigeria, a dichotomy between the *individual* and the *communal life* of a patient requiring palliative care emerged. In order to die in peace, the individual life of the patient needed completion.

FG1: *“Good dying is dying with one’s dreams accomplished*.*”*

Participants reported two important aspects of the completion of the individual life. First, seriously ill patients should die in the comfort of their own homes, amidst their own memories and personal objects

FG2/3: *“A good death is to die peacefully at home*.*”*

Second, seriously ill patients needed to be attuned to their god (or gods) either through making peace with these god(s), believing in them, or even fearing them.

FG2/3: *“Good dying is to die being reconciled with God and people*.*”*

With regard to good dying and/or a good death within a community, participants made a distinction in interactions between seriously ill patients and an inner circle of family and closest friends, interactions between dying patients and their wider communities, and interactions between dying patients and the Nigerian health care system. Participants explained that, in general, dying alone is considered bad dying whereas “dying in the home with relatives” (FG 1; “loved ones” FG 2; “surrounded by loving care” FG 3) is good dying. One precondition for dying surrounded by loving care is that patients acknowledge their loved ones.

FG1: *“Good dying depends on being able to say ‘I love you’ to people that matter*.*”*

Another precondition was that families and close friends come to terms with the approaching death of their beloved ones.

FG1: *“Family and friends have to assist [the patient] going on his/her journey*.*”*

According to participants, sharing information and education is key to preparing patients, families and friends for what is coming. The patient should be informed about the expected course of the disease and the potential occurrence of certain symptoms. The patient and the family should also be prepared for the economic consequences of having to take care of a seriously ill patient.

FG2/3: *“A good death is the consequence of a brief illness that leads to death without wasting family resources [and inheritance]*.*”*

The interactions between seriously ill patients and wider communities should, according to participants, be dominated by fulfilling one’s social lives. Patients have to sort out negative, troubled relationships with others and be forgiven if necessary.

FG1: *“A good death comes to those who can forgive all who offended them and also be forgiven before dying*.*”*

This sorting out of relationships with others is complicated by social stigmas, often related to having a serious illness. These stigmas isolate patients and family members from the community, taking away the Nigerian culture of living together with extended families (F G1: “We don’t leave them alone. What happens to them happens to us.”). Respondents stated that in the last decades migration has also interfered with this culture of extended families living together. As family members more frequently traveled to live abroad, family co-dependency was subjected to pressure. In general, these emigrants stay involved in patient care from a distance, for example by providing financial support.

For the focus group participants, receiving treatment for pain is central to the interaction between the seriously ill patient and the health care system. Health care, and appropriate medicaments like morphine in particular, should be available to and support patients until the end.

FG1: *“Good dying is dying with all modalities of care available*.*”*

Whether it is due to a failing health care system or to a cultural effort to increase social status, some of the more wealthy patients chose to abandon the comfort of the home “to be treated in a big hospital, preferably abroad” (FG2/3).

### Barriers to Palliative Care Provision in Nigeria

With regard to barriers to palliative care provision to achieve good dying and/or a good death, respondents referred to the socio-economic burden of palliative care. Moreover, taboos on dying of a life threatening illness that complicate providing information, restricted access to adequate medical-technical palliative care, attempts to reconcile hope based on religion (including miracle cure) with evidence-based care, and faulty implementation of palliative care policy by the government hampered good dying and/or a good death.

Due to the absence of a public social safety net and broad health insurance coverage, patients and families are facing a fee-for-service delivery system (“out of pocket payment”) that brings about a socio-economic paradox: with taking good care of a palliative patient, the burden on the patient’s social system will only increase. Taking care will consume time that would normally be spent on working in order to earn a living, whereas costs for medication persist. Respondents acknowledged that this mutual dependency of family’s resources and patient care, the family had a decisive say in care decisions. Furthermore, patients would always take the future well being of their families into consideration, up to a point where patients abandon further medical treatment to save future care costs.

Respondents believed in preparing patients and families through information, but information provision was complicated by taboos on life-threatening illnesses, dying and death. Nigerian palliative care providers have the delicate task to find out “how much patients want to know”, without confronting them with words such as ‘death’ or ‘dying’. CPCN said they try to inform the community (e.g., via churches or high-school students who relate to the younger children as well as to the elderly) about life-threatening illnesses, such as cancer, sickle cell, and HIV, in order for the community to recognize early symptoms of these illnesses, to identify patients eligible for palliative care, and to reduce stigmas. The relationship between HIV and palliative care changed radically the last decade due to the PEPFAR program. This program, sponsored by the American government, managed to turn HIV into a chronic disease in Nigeria through free provision of medication. The University College Hospital in Ibadan also followed a decree by the Nigerian government that patients with HIV/Aids should get free hospitalization. CPCN’s palliative care service now only sees HIV-patients who suffer from severe co morbidity or complex pains. Education of health care providers is their third goal, as many of these providers had a similar reluctance to talk about dying and death. They lacked both evidence based medical knowledge and communication skills to explain dying scenarios, and needed to learn about care opportunities in case a patient could no longer be cured.

With respect to palliative patients having access to all modalities of care, Nigerian palliative care has been complicated by the unavailability of and/or the lack of accessibility to opioids, which are necessary to treat severe pains. Moreover, CPCN’s palliative care team could only access medical technical equipment for pain management (e.g., morphine pump) in UCH’s ICU-unit. The hospice unit provided daycare for those who were able to come, including children. The team also visited outpatients who were confined to their sickbeds at home within a 30-kilometer radius around UCH and/or tried to provide follow up through phone. Patients who lived outside the 30-kilometer radius sometimes received up to a two-month provision of medicaments including oral morphine based on patients need. Follow up is provided through phone or by means of trained palliative care personnel in the area of patients’ domicile (“zonal coordinators”). However, many of such patients were often lost to follow up.

Focus groups, observations, and interviews showed that religion and spirituality were important themes in Nigerian palliative care. CPCN’s palliative care team started the day with a word of prayer, but team members were reluctant in mentioning their own religion in care settings. Instead, they used a neutral questionnaire to explore patients’ beliefs and tried to combine patients’ focus on their religion with conventional treatment. The conventional treatment then could apply to everyday quality of life whereas religion still nurtured hope for a miraculous cure.

Respondents referred to the existence of a national policy of palliative care as part of a national policy on cancer control. Many respondents, however, also mentioned a lack of policy implementation, diffusion and updates of medical knowledge, and government funding. Respondents acknowledged that Nigeria has a functioning health care service system at primary, secondary and tertiary levels, but that the system suffered from a lack of funding by government whereas many ‘no shows’ occurred due to the fee for service system; this lack of funding and patients was especially true for public primary care settings. Consequently, resources for palliative care were scarce or absent in primary care. More and more, UCH is becoming a tertiary care center providing both primary and geriatric care, while maintaining vivid alliances with satellite primary care facilities.

### Telemedicine in a Nigerian Care Context

With regard to opportunities and barriers for (future) telemedicine development in Nigeria, the unstructured interviews revealed technical, political, and socio-economic aspects. Many Nigerians, including patients and caregivers, possess mobile phones, whereas tablet computers are increasingly found among younger physicians. The presence of such hardware is a clear opportunity for some kinds of telemedicine. However, three basic technical requirements to use modern ICTs—bandwidth, network coverage, stable power supply—could not be met on a regular basis, thereby limiting telemedicine development. For example, 3G-networks were only available in urban areas. Moreover, there may be unexpected and/or long-term interruptions because of the low density of networks or disruptions in energy supply. Observations and interviews also exposed a few socio-economic aspects of telemedicine such as theft of valuable communication technologies equipment and international medical tourism that received a boost from international teleconsultations.

## Discussion

### Nigerian Conceptions of Good Dying in Light of Prior African Studies on Good Dying

This study’s results on good dying and good death in Nigeria are, for the most part, consistent with other literature on good dying and good death-concepts in sub-Saharan Africa. This similarity of death perceptions across cultures has been noticed before [40]. A good death in Nigeria, like in other parts of Africa, is a death that ‘completes’ the individual life [40,41]. A good death comes naturally and without human interference, and is the result of “successful aging” [40]. A good death also contains fulfillment of a spiritual and/or religious life [42]. Nigerian health care professionals expressed their desire to parallel regular palliative care with a religion that provides hope for a miraculous cure. However, they admitted, in line with the literature [40–43], that religion and traditional beliefs strongly influence people’s perceptions on dying and death and complicate palliative care.

Nigerian health care professionals, like other sub-Saharan Africans, consider home to be the preferred place of death because of its peacefulness, the personal memories, and the presence of loving family and friends [10,40,44]. At home, patients can still try to be of value to their family members [41]. A desire to die at home is often also motivated by socio-economic reasons as travel to and staying in a hospital is usually considered too costly and/or too disruptive to daily life responsibilities [44,45]. However, in some sub-Saharan countries (e.g., Namibia) the public preference concerning place of death inclined towards the hospital [46]. A potential explanation could be the low-level home palliative care coverage and/or distrust of the local expertise [10,45–47].

Costs for care are a major issue in Nigerian palliative care, just like in the rest of sub-Saharan Africa. With the lack of a public social safety net and/or broad health insurances, Nigerian palliative care patients have to pay fees for services, including receiving appropriate analgesics [41], food [43,45], or a proper bed. Due to the costs of professional care, patients usually have to rely heavily on family care and family money [40]. In this way, a vicious circle is created in which both severely ill patients and family caregivers lose their income because of lack of energy or lack of time, inducing family poverty and an increasing lack of care resources [10,41,44]. Nigerian patients, according to the health professionals, often desired not to be a financial burden to their family [41] and were even willing to let the family decide for their care.

To realize good dying, Nigerian patients, families, and friends have to come to terms with the patient being seriously ill [40]. Education of patients and families is considered essential in Nigeria and in the rest of sub-Saharan Africa so that they could accept the serious situation and prepare for it [41,46]. Education for the wider community is thought to reduce seriously ill patients’ and their families’ suffering from social stigma [10,41,43]. With respect to patients, Nigerian health care professionals had to find out how much they wanted to know without talking about dying and death directly. Other studies in Africa show that people are open to information about incurability, care options, and future symptoms and problems. They just do not want to be informed about prognosis [41,47]. Like in the rest of sub-Saharan Africa, the Nigerian health care professionals noticed that (fear of) pain is the most important issue for patients [44]. Appropriate drugs and strong opioids, however, are often unavailable to patients [43].

### The Fit of Telemedicine in a Nigerian Palliative Care Practice

Whether and how does telemedicine for palliative care fit the Nigerian socio-cultural context with its particular health care needs and local cultural expectations towards dying [30]? Theoretically, telemedicine can provide palliative care tele-education, options for teleconsultation, or information databases [17,18,20]. It could improve palliative care provision by community health care professionals and bring quality care closer to home, thereby keeping families more intact and social and spiritual fulfillment more within reach [44]. Chances that patients are surrounded by a loving family and caring community throughout the course of the disease increase with well-trained local health care professionals who are able to fit palliative care into local community and to sensitively address taboos and/or stigma’s [8–10,42]. However, low-threshold telemedicine cannot be realized as long as Internet bandwidth, network coverage and power supply are not guaranteed. Both tele-education and teleconsultation strongly depend on limitedly available interactivity. Moreover, important palliative care information might not be available when needed due to a lack of access to the Internet. Nigerian funds to set up new, but not too eye-catching projects with stable and easily accessible technologies are hard to find.

Based on this research’ data, the Dutch researchers (JG, JH) together with the CPCN representatives (OS, KO, SD) discussed and formulated a feasibility plan for future telemedicine development in a Nigerian palliative care context. Two scenarios for further development were formulated (Table 2). In the first, futuristic scenario, existing and new care networks between UCH and satellite primary care facilities (e.g., PEPFAR, geriatrics) could be enriched with real-time audiovisual tele*consultations* for palliative care. The advantages of this scenario are that patients can be seen on a regular basis while staying relatively close to home. As a side benefit, the attending primary care physicians receive specific education about palliative care. Current Internet and /or electricity connections are not yet stable enough to enable reliable real-time video connections outside the city centre. Satellite connections could solve these instabilities, but these come at significantly higher costs. A ‘lite’ tele*consultation* version includes the use of mobile phones, which enable synchronous telephone calls and text messages (e.g., Whatsapp), or non-synchronous sharing of photo’s by e-mail. Mobile phones could also be used to support and educate patients’ family caregivers (mHealth); an initiative that already has been taken up by CPCN.

**Table 2:** Scenarios for applying telemedicine in Nigerian palliative care services.

	*Professional development*	*Costs of technology*	*Integration with care networks*	*Sustainability*
Teleconsultation	++	—	++	?
‘Lite’ Teleconsultation	+	+/-	+	+
mHealth directly with patients	-	+	—	+
Tele-education	++	—	+/-	?
‘Lite’ Tele-education	++	+/-	+	+

***Key***

++: significant positive impact

+: positive impact

+/-: equivocal impact

-: negative impact

—: significant negative impact

?: as of yet unknown

The second scenario focuses on building a tele-*educational* network between CPCN and professionals interested in the project. With the experience of the face-to-face palliative care training courses, CPCN could develop a nation-wide e-learning program on palliative care. Interactive case discussions and lectures can be set up via online streaming although this streaming again requires stable high-end internet connections. A ‘lite’ version, however, could include national and international online lectures via public streaming channels, and non-synchronous chat sessions on a central website. One of the advantages is that the tele-education scenario enhances the educational activities of the CPCN and contributes to the development of a nationwide network of professionals keen to undertake continuous palliative care education. Moreover, such an e-learning tool can easily be expanded with content for third parties such as community leaders, schoolteachers, or religious leaders. As broadband Internet penetration increases and quality of service improves synchronous interactivity could be added on to the platform.

Only the ‘lite’ tele-*educational* scenario seems viable in Nigeria, building on non-synchronous communication between specialist palliative care centers and community health care providers to engage in necessary educational activities against reasonable costs and low threshold technology [4]. This ‘lite’ version could potentially be expanded towards a full, interactive version when the technology and network coverage allow for this. The ‘lite’ tele-educational scenario could build on already existing care networks, for example primary care networks or the network of PEPFAR and facilitate a bottom up, grassroots approach to palliative care implementation in the community.

### Future Research

Future research in the area of telemedicine in Nigeria largely depends on the future availability of funds for low-key development and evaluations of *tele-education* modules. In collaboration with the available health care networks, the thoroughly evaluated web-based learning modules could be extended with synchronous teleconsultation on the condition that the Internet networks of Nigeria and other sub-Saharan African countries keep expanding and improving. In the final stage of development, patients could be involved. For now, future research should therefore focus on a) how to develop asynchronous web-based learning modules that suit the Nigerian (or sub-Saharan) context, b) what will be opportunities and barriers to implement these e-learning modules, and c) how such e-learning modules improve professional capacity and, down the line, patient’ and family caregivers’ outcomes [4].
